# Osseous Scaphotrapezial Coalition

**DOI:** 10.1155/2015/345351

**Published:** 2015-12-09

**Authors:** William M. Weathers, Susanna C. Spence, Nicholas M. Beckmann

**Affiliations:** Department of Radiology, University of Texas Health Science Center Houston, 6431 Fannin Street 2.130B, Houston, TX 77039, USA

## Abstract

Osseous scaphotrapezial coalition is one of the rarest forms of carpal coalition of the hand. Often discovered incidentally, pain and functional limitation have not been reported. Carpal coalitions occurring across the carpal rows are thought to occur as a result of some insult or congenital anomaly. Isolated scaphotrapezial coalition calls into question the traditional thinking that fusion between the proximal and distal carpal rows must be acquired or associated with congenital syndromes.

## 1. Introduction

Carpal coalition is a bony, fibrous, or cartilaginous connection between adjacent carpal bones [1]. It is a rare anomaly reported to occur in up to 0.1% to 9% of the population with a higher incidence in females and people of African descent [2]. Patients are typically asymptomatic, and the entity is often found incidentally during work-up for trauma or unrelated hand and wrist pain [3]. Lunotriquetral coalition is the most common form followed by capitohamate coalition [3–5]. Of the carpal coalitions, scaphotrapezial coalition is one of the rarest forms. In this paper, we present three cases of asymptomatic osseous scaphotrapezial coalition.

## 2. Case Series

### 2.1. Case 1

**Figure 1:**
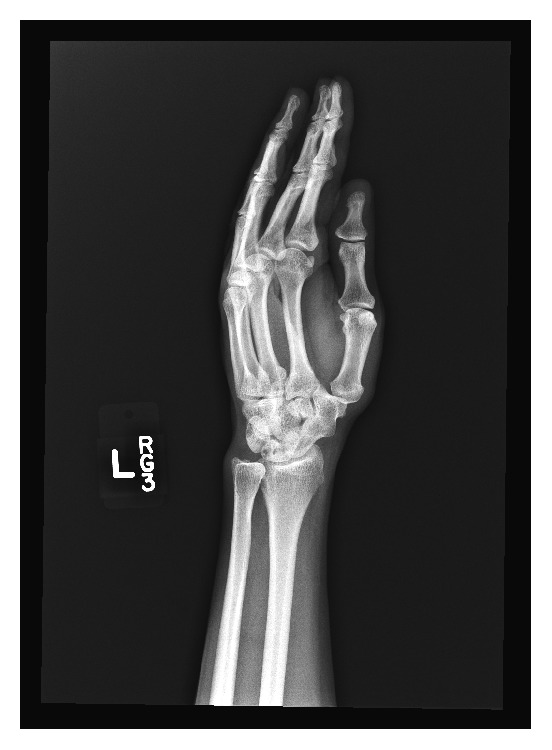
Oblique radiograph of the left wrist in patient 1 demonstrates osseous carpal coalition of the scaphoid and trapezium.

A 55-year-old white male presented to an outpatient clinic for chronic left wrist pain. The pain had been present off and on for 5–10 years and was worst with doing push-ups. The patient reported multiple falls in the past for which he did not seek medical care. Otherwise, he had no contributory past medical history. Plain film evaluation of the wrist and hand was obtained and demonstrated sclerosis and cystic changes of the lunate consistent with osteonecrosis of the lunate. An incidental finding of scaphotrapezial coalition was noted (Figure 1).

### 2.2. Case 2

**Figure 2:**
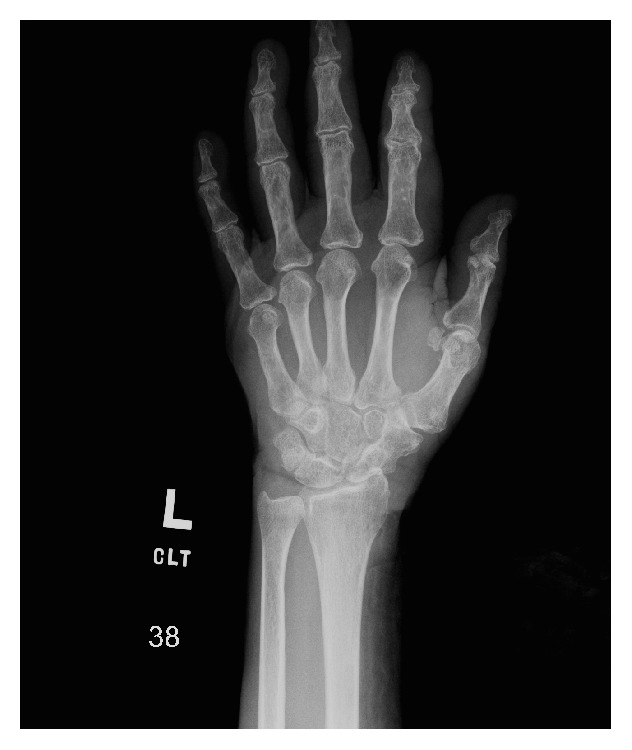
Frontal radiograph of patient 2 demonstrates isolated osseous scaphotrapezial coalition.

A 77-year-old African-American female presented to the emergency room with bilateral wrist pain and swelling after a fall from bed. Her past medical history was noncontributory. Radiographs of the hands were obtained, which demonstrated no acute injury. Scaphotrapezial coalition was noted in both wrists (Figure 2).

### 2.3. Case 3

**Figure 3:**
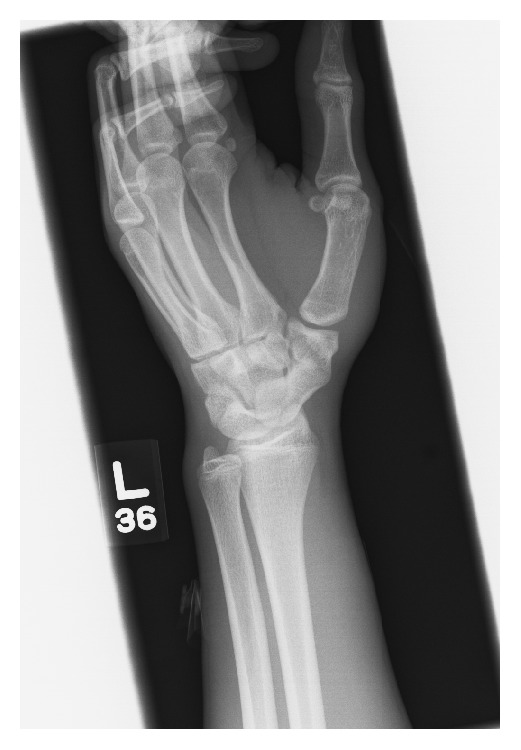
Oblique radiograph of the wrist demonstrates isolated scaphotrapezial coalition.

The patient is a 16-year-old African-American male that presented to the emergency room after a high speed motor vehicle collision. The patient suffered a right clavicle fracture and left ulna fracture. During trauma work-up, radiographs of the left wrist were obtained. No acute abnormality of the wrist was seen. Incidental note of a scaphotrapezial coalition in the left wrist was made (Figure 3).

## 3. Discussion

This case series was reported given the paucity of the scaphotrapezial type osseous carpal coalitions reported in the literature. All of our patients' coalitions were discovered incidentally, and no direct link between wrist pain and the coalition could be ascertained. In case 1, the patient's pain was likely a result of the osteonecrosis of the lunate. The other two patients' coalitions were discovered incidentally after a trauma work-up. Two of our patients were African-American and one of the patients had bilateral scaphotrapezial coalition; however, bilateral wrist radiographs were only obtained in one patient. These findings are similar to those observed in the literature, with increased incidence in African-American people and the presence of bilaterality [3, 6, 7].

Carpal coalition occurs as a result of failure or incomplete cavitation of the cartilaginous carpal precursor during fetal life [8]. The term fusion is to be avoided, since this is a failure of segmentation during development and not a joining of two distinct structures [2, 7, 9, 10]. Coalition occurring as a result of infection, trauma, or inflammatory etiologies is more accurately described as fusion or ankylosis. Our cases of osseous coalition are likely congenital in nature as no significant trauma, infectious, or inflammatory etiology was present.

In general, osseous carpal coalition is a rare incidentally noted asymptomatic phenomenon seen on plain film radiographs. The true incidence of carpal coalition is likely underreported, as fibrous and cartilaginous types of carpal coalition are not readily apparent on plain film radiography—which is the overwhelming imaging modality for work-up of wrist pain. Majority of the cases of carpal coalition represent mere academic curiosities rather than clinically significant findings. However, carpal coalition has been associated with multiple syndromes including arthrogryposis, symphalangia, hand-foot-uterus, Ellis-van Creveld, Holt-Oram, otopalatodigital, diastrophic dwarfism, Turner's, and dyschondrosteosis [11].

Historically, scaphotrapezial coalition was thought to occur in conjunction with congenital syndromes. It was generally accepted that coalitions bridging the proximal and distal carpal rows and involving multiple carpal bones were more likely to be found in patients with acquired carpal abnormalities or congenital syndromes [11]. In contrast, coalitions occurring in the same carpal row were more likely to be isolated and incidental carpal anomalies. Scaphotrapezial coalition has been associated with multiple syndromes including otopalataldigital syndrome, symphalangism, and hand-foot-uterus syndrome [11–13]. Recently, more cases, including this case series, have found scaphotrapezial coalition as an incidental finding during work-ups for hand pain without an associated congenital syndrome or specific etiology.
